# Crystal structure of disodium 2-amino-6-oxo-6,7-di­hydro-1*H*-purine-1,7-diide hepta­hydrate

**DOI:** 10.1107/S2056989015003163

**Published:** 2015-02-18

**Authors:** Dvir Gur, Linda J. W. Shimon

**Affiliations:** a Department of Structural Biology, Weizmann Institute of Science, 76100 Rehovot, Israel; b Chemical Research Support, Weizmann Institute of Science, 76100 Rehovot, Israel

**Keywords:** crystal structure, guanine salt, nucleobase, hydrogen bonds

## Abstract

In the title compound, disodium 2-amino-6-oxo-6,7-di­hydro-1*H*-purine-1,7-diide hepta­hydrate, 2Na^+^·C_5_H_3_N_5_O^2−^·7H_2_O, the structure is composed of alternating (100) layers of guanine mol­ecules and hydrated Na^+^ ions. Within the guanine layer, the mol­ecules are arranged in centrosymmetric pairs, with a partial overlap between the guanine rings. In this compound, guanine exists as the amino–keto tautomer from which deprotonation from N_1_ and N_7_ has occurred (purine numbering). There are no direct inter­actions between the Na^+^ cations and the guanine anions. Guanine mol­ecules are linked to neighboring water mol­ecules by O—H⋯N and O—H⋯O hydrogen bonds into a network structure.

## Abstract 2

In the title compound, the deprotonated guanine mol­ecules are arranged in centrosymmetric pairs, and form hydrogen bonds with the neighboring water mol­ecules.

## Chemical context   

Guanine is one of the five nucleic acids present in both DNA and RNA (Blackburn *et al.*, 2006 ▸), and is also found in its crystalline form in the integument of many animals as a light reflector (Land, 1972 ▸; Parker, 2000 ▸; Gur *et al.*, 2013 ▸, 2014 ▸). There are two known crystal structures of guanine; guanine monohydrate (Thewalt *et al.*, 1971 ▸) and anhydrous guanine (Guille & Clegg, 2006 ▸). In addition there are also a few known guanine salts (Broomhead, 1951 ▸; Wei, 1977 ▸; Iball & Wilson, 1965 ▸). The crystal structure of the title compound was obtained as a part of a study into controlling the crystal phase of guanine using recrystallization.
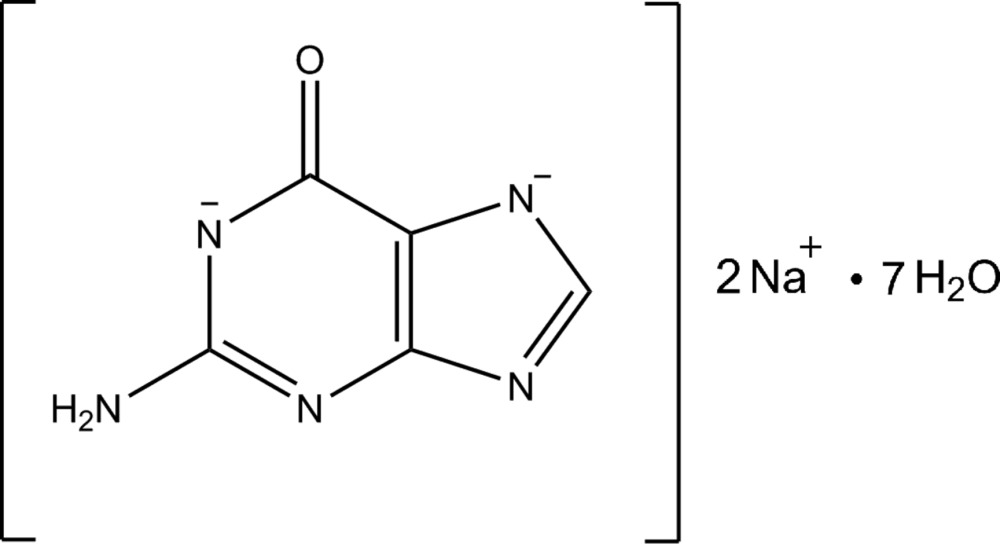


Cation, anion and radical formation among nucleic acids are thought to be important steps in DNA damage (Cooke *et al.*, 2003 ▸; Kasai, 1997 ▸). For that reason, protonation and deprotonation of nucleic acids and their role in processes like mutation has been widely studied both theoretically and experimentally. It is thought that the most prominent site for this kind of damage will be guanine because it has the lowest oxidation potential among the four DNA bases (Burrows & Muller, 1998 ▸; Steenken & Jovanovic, 1997 ▸). As a result, even initially different oxidized species may eventually migrate to guanine. Therefore, DNA damage is predicted to be produced at this site (Melvin *et al.*, 1995 ▸). The crystal structure of the deprotonated guanine presented in this report may provide information about the deprotonated oxidized guanine state and its inter­actions with the neighboring water mol­ecules.

## Structural commentary   

**Figure 1:**
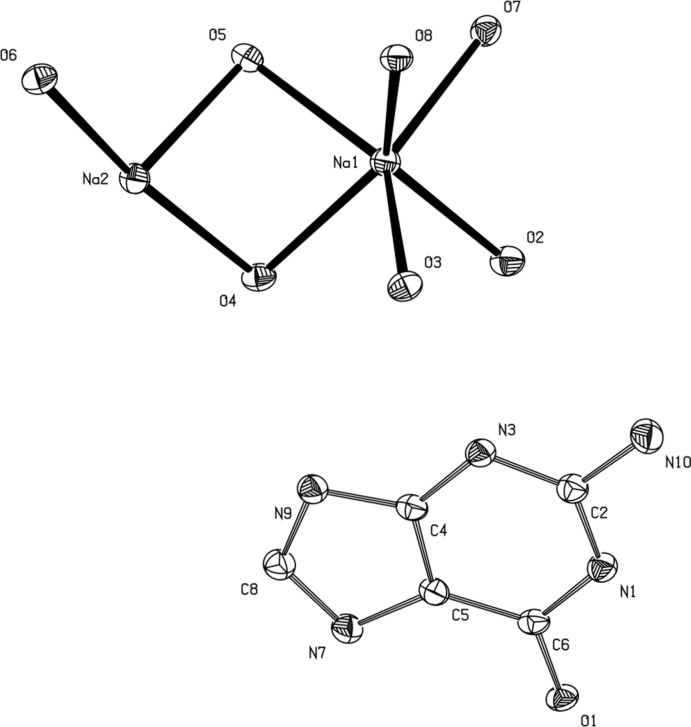
A displacement ellipsoid plot of the asymmetric unit drawn at the 50% probability level. H atoms have been omitted for clarity.

In the structure of the title compound, the asymmetric unit is composed of a guanine anion, two sodium counter-ions and seven water mol­ecules (Fig. 1 ▸). In this compound, guanine exists as the amino–keto tautomer, the guanine mol­ecules are doubly negatively charged, as a result of the deprotonation from N1 and N7 (purine numbering) that occurred due to the alkaline conditions of the solution from which recrystallization took place. There are no direct inter­actions between the Na^+^ cations and the guanine anions.

## Supra­molecular features   

**Figure 2:**
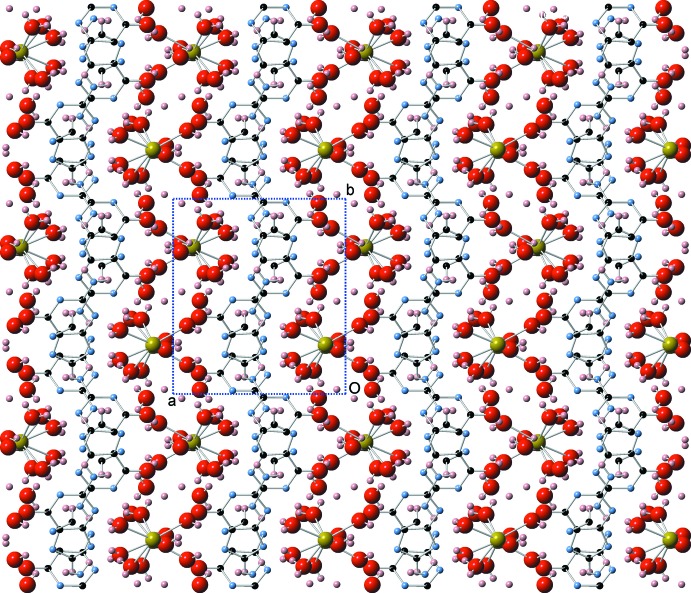
The crystal structure viewed down the *c* axis, showing the alternating layers of guanine mol­ecules and hydrated sodium ions.

**Figure 3:**
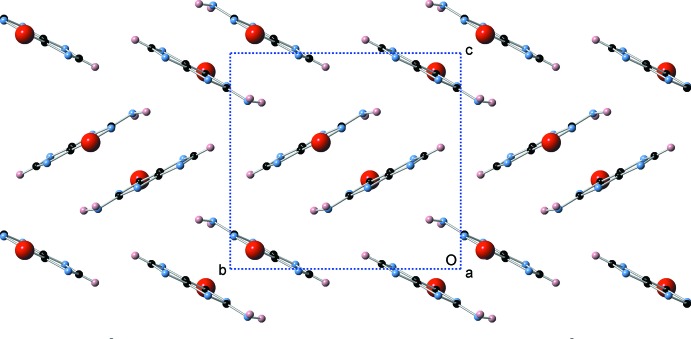
A view down the *a* axis showing the herringbone crystal packing motif, including edge-to-face inter­actions between the guanine dimers.

The structure is composed of alternating (100) layers of guanine mol­ecules and hydrated Na^+^ Ions (Fig. 2 ▸). Within the guanine layer, the mol­ecules are arranged in centrosymmetric pairs, in which a partial overlap between the guanine rings is present. The distances between the overlapping atoms C_2_–N_3_^i^ and C_4_–N_10_^i^ are 3.415 (2) and 3.460 (2) Å, respectively [symmetry code: (i) = 1 − *x*, 1 − *y*, 1 − *z*]. The two mol­ecules are offset presumably to separate the charged N^−^ ions of the two mol­ecules and at the same time provide van der Waals contacts between the two rings. In most known guanine crystal structures, neighboring guanine mol­ecules form hydrogen bonds that result in flat layers of guanine mol­ecules, between which stacking inter­actions are present. Such layers are not present in the structure of the title compound. Instead, the guanine mol­ecules form O—H⋯N and O—H⋯O hydrogen bonds with the neighboring water mol­ecules (Table 1 ▸), satisfying all guanine donors and acceptors with the exception of the NH_2_ amine group, which surprisingly does not seem to participate in any hydrogen bonding, and is not within hydrogen-bonding distance of any hydrogen acceptors. In addition, the guanine mol­ecules form dimers that have an edge-to-face type orientation, resulting in the observed herringbone crystal packing motif with a dihedral angle of 123.917 (17)° (Fig. 3 ▸).

## Synthesis and crystallization   

Disodium 2-amino-6-oxo-6,7-di­hydro-1*H*-purine-1,7-diide hepta­hydrate was prepared by dissolving 0.1 g guanine (powder Sigma–Aldrich) in 5 ml NaOH 1 *N* (pH 14). The solution was then filtered using a PVDF filter (0.22 µm), and 0.1 ml of NaOH 1 *N* was added to the solution to ensure that all of the guanine was dissolved. The solution was then kept for 10 days under an IR lamp using 15 min. cycles (on/off) while open to the atmosphere. Large 3mm crystals were extracted from the suspension, broken to a suitable size and subjected to single crystal X-ray diffraction.

## Refinement   

Crystal data, data collection and structure refinement details are summarized in Table 2 ▸. All hydrogen atoms were refined freely with the exception of C8-bound H atom that was placed in a calculated position and refined in riding mode.

## Supplementary Material

Crystal structure: contains datablock(s) I. DOI: 10.1107/S2056989015003163/pk2539sup1.cif

Structure factors: contains datablock(s) I. DOI: 10.1107/S2056989015003163/pk2539Isup2.hkl

CCDC reference: 1049453

Additional supporting information:  crystallographic information; 3D view; checkCIF report

## Figures and Tables

**Table 1:** Hydrogen-bond geometry (, )

*D*H*A*	*D*H	H*A*	*D* *A*	*D*H*A*
O2H2*A*N9^i^	0.84(3)	1.97(3)	2.7875(19)	168(3)
O2H2*B*N3	0.89(3)	2.08(3)	2.9582(19)	167(2)
O3H3*A*O5^ii^	0.87(3)	2.08(3)	2.9200(18)	163(3)
O3H3*B*N3	0.87(3)	1.95(3)	2.8038(18)	166(3)
O4H4*A*N1^iii^	0.85(3)	1.96(3)	2.8093(19)	177(3)
O4H4*B*N9	0.85(3)	2.14(3)	2.9866(19)	176(2)
O5H5*C*O1^iii^	0.81(3)	1.96(3)	2.7581(18)	168(3)
O6H6*A*O2^iv^	0.79(3)	2.02(3)	2.7938(19)	167(3)
O6H6*B*N7^v^	0.90(3)	2.01(3)	2.909(2)	173(2)
O7H7*A*O1^v^	0.88(3)	1.95(3)	2.7867(17)	160(3)
O7H7*B*O3	0.85(3)	1.92(3)	2.7608(18)	168(3)
O8H8*A*O1^iii^	0.84(3)	1.99(3)	2.8303(17)	171(3)
O8H8*B*N7^vi^	0.82(3)	1.98(3)	2.7938(19)	171(3)
O5H5*D*O1^vii^	0.78(3)	2.02(3)	2.7835(17)	164(3)

Symmetry codes: (i) 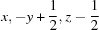; (ii) 
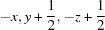
; (iii) 
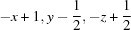
; (iv) 
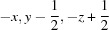
; (v) 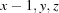; (vi) 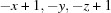; (vii) 
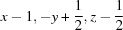
.

**Table 2:** Experimental details

Crystal data	
Chemical formula	2Na^+^C_5_H_3_N_5_O^2^7H_2_O
*M*_r_	321.21
Crystal system, space group	Monoclinic, *P*2_1_/*c*
Temperature (K)	120
*a*, *b*, *c* ()	10.5520(2), 11.6936(3), 11.1938(2)
()	101.5758(13)
*V* (^3^)	1353.12(5)
*Z*	4
Radiation type	Mo *K*
(mm^1^)	0.20
Crystal size (mm)	0.30 0.10 0.05

Data collection	
Diffractometer	Nonius KappaCCD
Absorption correction	Multi-scan (*DENZO-SMN*; Otwinowski Minor, 2006 ▸)
*T*_min_, *T*_max_	0.977, 0.990
No. of measured, independent and observed [*I* > 2(*I*)] reflections	6648, 3931, 2981
*R*_int_	0.019
(sin /)_max_ (^1^)	0.704

Refinement	
*R*[*F* ^2^ > 2(*F* ^2^)], *wR*(*F* ^2^), *S*	0.049, 0.147, 1.07
No. of reflections	3931
No. of parameters	248
H-atom treatment	H atoms treated by a mixture of independent and constrained refinement
_max_, _min_ (e ^3^)	0.57, 0.39

Computer programs: *COLLECT* (Nonius, 1998 ▸), *DENZO-SMN* (Otwinowski Minor, 2006 ▸), *SHELXS97* (Sheldrick, 2008 ▸), *SHELXL2013* (Sheldrick, 2015 ▸), *PLATON* (Spek, 2009 ▸), *CrystalMaker* (CrystalMaker, 2010 ▸) and *publCIF* (Westrip, 2010 ▸).

## supplementary crystallographic information

### Crystal data

**Table:**

2Na^+^·C_5_H_3_N_5_O^2^^−^·7H_2_O	*F*(000) = 672
*M**_r_* = 321.21	*D*_x_ = 1.577 Mg m^−^^3^
Monoclinic, *P*2_1_/*c*	Mo *K*α radiation, λ = 0.71073 Å
*a* = 10.5520 (2) Å	Cell parameters from 3810 reflections
*b* = 11.6936 (3) Å	θ = 2.6–30.0°
*c* = 11.1938 (2) Å	µ = 0.20 mm^−^^1^
β = 101.5758 (13)°	*T* = 120 K
*V* = 1353.12 (5) Å^3^	Plate, colourless
*Z* = 4	0.30 × 0.10 × 0.05 mm

### Data collection

**Table:**

Nonius KappaCCD diffractometer	2981 reflections with *I* > 2σ(*I*)
Radiation source: sealed tube	*R*_int_ = 0.019
φ and ω scans	θ_max_ = 30.0°, θ_min_ = 3.7°
Absorption correction: multi-scan (*DENZO-SMN*; Otwinowski & Minor, 2006)	*h* = −14→14
*T*_min_ = 0.977, *T*_max_ = 0.990	*k* = −12→16
6648 measured reflections	*l* = −15→15
3931 independent reflections	

### Refinement

**Table:**

Refinement on *F*^2^	0 restraints
Least-squares matrix: full	Hydrogen site location: difference Fourier map
*R*[*F*^2^ > 2σ(*F*^2^)] = 0.049	H atoms treated by a mixture of independent and constrained refinement
*wR*(*F*^2^) = 0.147	*w* = 1/[σ^2^(*F*_o_^2^) + (0.0862*P*)^2^ + 0.5094*P*] where *P* = (*F*_o_^2^ + 2*F*_c_^2^)/3
*S* = 1.07	(Δ/σ)_max_ < 0.001
3931 reflections	Δρ_max_ = 0.57 e Å^−^^3^
248 parameters	Δρ_min_ = −0.39 e Å^−^^3^

### Special details

**Table:**

Geometry. All e.s.d.'s (except the e.s.d. in the dihedral angle between two l.s. planes) are estimated using the full covariance matrix. The cell e.s.d.'s are taken into account individually in the estimation of e.s.d.'s in distances, angles and torsion angles; correlations between e.s.d.'s in cell parameters are only used when they are defined by crystal symmetry. An approximate (isotropic) treatment of cell e.s.d.'s is used for estimating e.s.d.'s involving l.s. planes.

### Fractional atomic coordinates and isotropic or equivalent isotropic displacement parameters (Å^2^)

**Table:**

	*x*	*y*	*z*	*U*_iso_*/*U*_eq_	
N1	0.65524 (13)	0.48287 (13)	0.34832 (13)	0.0172 (3)	
C2	0.52557 (16)	0.48307 (15)	0.33930 (15)	0.0175 (3)	
N3	0.45252 (13)	0.40241 (13)	0.37665 (13)	0.0170 (3)	
C4	0.52344 (15)	0.31289 (15)	0.43267 (14)	0.0158 (3)	
C5	0.65775 (15)	0.30526 (15)	0.44968 (14)	0.0161 (3)	
C6	0.72597 (15)	0.39376 (14)	0.40446 (14)	0.0156 (3)	
N7	0.69798 (13)	0.20349 (13)	0.50957 (13)	0.0186 (3)	
C8	0.58601 (16)	0.15733 (16)	0.52393 (16)	0.0193 (3)	
H8	0.585 (2)	0.087 (2)	0.563 (2)	0.023*	
N9	0.47610 (13)	0.21804 (13)	0.47962 (13)	0.0178 (3)	
N10	0.46123 (16)	0.57498 (15)	0.27867 (15)	0.0235 (3)	
H10A	0.378 (3)	0.585 (3)	0.290 (3)	0.057 (9)*	
H10B	0.513 (3)	0.636 (3)	0.271 (3)	0.048 (8)*	
O1	0.85043 (11)	0.39326 (11)	0.41331 (10)	0.0171 (3)	
Na1	0.11549 (6)	0.25431 (6)	0.18356 (6)	0.01689 (17)	
Na2	0.04858 (6)	0.04502 (6)	0.37257 (6)	0.01704 (17)	
O2	0.30698 (12)	0.32592 (11)	0.13607 (12)	0.0194 (3)	
H2A	0.355 (3)	0.303 (3)	0.090 (3)	0.043 (8)*	
H2B	0.362 (3)	0.346 (2)	0.204 (2)	0.031 (6)*	
O3	0.18432 (12)	0.38036 (11)	0.35525 (11)	0.0186 (3)	
H3A	0.143 (3)	0.445 (3)	0.352 (3)	0.048 (8)*	
H3B	0.267 (3)	0.391 (3)	0.374 (3)	0.042 (7)*	
O4	0.24329 (12)	0.11485 (11)	0.32009 (11)	0.0177 (3)	
H4A	0.276 (3)	0.075 (2)	0.271 (3)	0.035 (7)*	
H4B	0.307 (3)	0.146 (2)	0.367 (2)	0.032 (7)*	
O5	−0.00208 (13)	0.06838 (11)	0.14535 (11)	0.0174 (3)	
H5C	0.050 (3)	0.024 (3)	0.128 (3)	0.042 (8)*	
H5D	−0.051 (3)	0.089 (3)	0.087 (3)	0.040 (7)*	
O6	−0.15505 (12)	0.02121 (12)	0.42404 (11)	0.0200 (3)	
H6A	−0.207 (3)	−0.027 (3)	0.401 (3)	0.037 (7)*	
H6B	−0.203 (3)	0.079 (2)	0.444 (2)	0.037 (7)*	
O7	0.04292 (13)	0.22690 (11)	0.46296 (11)	0.0180 (3)	
H7A	−0.030 (3)	0.265 (3)	0.447 (3)	0.041 (7)*	
H7B	0.094 (3)	0.274 (2)	0.440 (3)	0.034 (7)*	
O8	0.08223 (12)	−0.14985 (11)	0.31485 (11)	0.0175 (3)	
H8A	0.108 (2)	−0.143 (2)	0.249 (3)	0.033 (7)*	
H8B	0.145 (3)	−0.173 (2)	0.365 (3)	0.034 (7)*	

### Atomic displacement parameters (Å^2^)

**Table:**

	*U*^11^	*U*^22^	*U*^33^	*U*^12^	*U*^13^	*U*^23^
N1	0.0152 (6)	0.0196 (7)	0.0171 (6)	−0.0014 (5)	0.0042 (5)	0.0002 (5)
C2	0.0158 (7)	0.0205 (8)	0.0162 (7)	0.0002 (6)	0.0034 (6)	−0.0002 (6)
N3	0.0139 (6)	0.0197 (7)	0.0172 (6)	−0.0007 (5)	0.0030 (5)	0.0006 (5)
C4	0.0130 (7)	0.0214 (8)	0.0127 (7)	0.0011 (6)	0.0023 (5)	−0.0006 (6)
C5	0.0141 (7)	0.0201 (8)	0.0140 (7)	0.0015 (6)	0.0024 (5)	0.0016 (6)
C6	0.0131 (7)	0.0207 (8)	0.0128 (7)	−0.0008 (6)	0.0021 (5)	−0.0024 (6)
N7	0.0154 (6)	0.0205 (7)	0.0194 (7)	0.0015 (5)	0.0022 (5)	0.0026 (5)
C8	0.0163 (8)	0.0220 (9)	0.0196 (8)	0.0012 (6)	0.0033 (6)	0.0020 (6)
N9	0.0154 (6)	0.0209 (7)	0.0169 (6)	0.0000 (5)	0.0030 (5)	0.0016 (5)
N10	0.0180 (7)	0.0243 (8)	0.0282 (8)	0.0031 (6)	0.0045 (6)	0.0072 (6)
O1	0.0106 (5)	0.0232 (6)	0.0177 (5)	−0.0012 (4)	0.0034 (4)	−0.0005 (4)
Na1	0.0157 (3)	0.0191 (4)	0.0157 (3)	−0.0004 (2)	0.0028 (2)	0.0002 (2)
Na2	0.0166 (3)	0.0193 (3)	0.0158 (3)	0.0003 (2)	0.0045 (2)	−0.0001 (2)
O2	0.0159 (6)	0.0261 (7)	0.0167 (6)	−0.0009 (5)	0.0045 (5)	−0.0018 (5)
O3	0.0135 (6)	0.0207 (6)	0.0223 (6)	0.0008 (5)	0.0047 (4)	−0.0024 (5)
O4	0.0134 (5)	0.0220 (6)	0.0176 (6)	0.0002 (5)	0.0030 (4)	−0.0028 (5)
O5	0.0166 (6)	0.0202 (6)	0.0146 (5)	0.0028 (5)	0.0013 (4)	0.0013 (5)
O6	0.0159 (6)	0.0230 (7)	0.0213 (6)	−0.0008 (5)	0.0043 (5)	−0.0027 (5)
O7	0.0171 (6)	0.0198 (6)	0.0177 (6)	0.0017 (5)	0.0049 (4)	0.0003 (4)
O8	0.0147 (6)	0.0229 (6)	0.0147 (5)	0.0016 (5)	0.0022 (4)	0.0012 (4)

### Geometric parameters (Å, º)

**Table:**

N1—C2	1.352 (2)	Na2—O7	2.3612 (14)
N1—C6	1.360 (2)	Na2—O4	2.3912 (14)
C2—N3	1.337 (2)	Na2—O8	2.4142 (15)
C2—N10	1.375 (2)	Na2—O6^iii^	2.4534 (14)
N3—C4	1.364 (2)	Na2—O5	2.5067 (14)
C4—N9	1.364 (2)	Na2—Na2^iii^	3.3871 (13)
C4—C5	1.394 (2)	Na2—Na1^iv^	3.8095 (9)
C5—N7	1.390 (2)	Na2—Na1^v^	4.1397 (9)
C5—C6	1.411 (2)	O2—H2A	0.84 (3)
C6—O1	1.2970 (19)	O2—H2B	0.89 (3)
N7—C8	1.338 (2)	O3—H3A	0.87 (3)
C8—N9	1.365 (2)	O3—H3B	0.87 (3)
C8—H8	0.94 (2)	O4—H4A	0.85 (3)
N10—H10A	0.91 (3)	O4—H4B	0.85 (3)
N10—H10B	0.91 (3)	O5—H5C	0.81 (3)
Na1—O2	2.3447 (14)	O5—H5D	0.78 (3)
Na1—O8^i^	2.3715 (14)	O6—Na2^iii^	2.4534 (14)
Na1—O3	2.4153 (14)	O6—H6A	0.79 (3)
Na1—O7^ii^	2.4440 (14)	O6—H6B	0.90 (3)
Na1—O4	2.4467 (14)	O7—Na1^v^	2.4440 (14)
Na1—O5	2.4972 (14)	O7—H7A	0.88 (3)
Na1—Na2	3.4006 (9)	O7—H7B	0.85 (3)
Na1—Na2^i^	3.8095 (9)	O8—Na1^iv^	2.3716 (14)
Na1—Na2^ii^	4.1397 (9)	O8—H8A	0.84 (3)
Na2—O6	2.3502 (14)	O8—H8B	0.82 (3)
			
C2—N1—C6	119.30 (14)	O4—Na2—O5	74.47 (4)
N3—C2—N1	127.87 (15)	O8—Na2—O5	81.04 (5)
N3—C2—N10	116.55 (15)	O6^iii^—Na2—O5	160.17 (5)
N1—C2—N10	115.49 (15)	O6—Na2—Na2^iii^	46.41 (3)
C2—N3—C4	112.80 (14)	O7—Na2—Na2^iii^	83.17 (4)
N3—C4—N9	126.26 (14)	O4—Na2—Na2^iii^	137.16 (5)
N3—C4—C5	124.10 (15)	O8—Na2—Na2^iii^	91.01 (4)
N9—C4—C5	109.64 (14)	O6^iii^—Na2—Na2^iii^	43.93 (3)
N7—C5—C4	108.93 (14)	O5—Na2—Na2^iii^	148.16 (5)
N7—C5—C6	132.26 (14)	O6—Na2—Na1	123.50 (4)
C4—C5—C6	118.80 (15)	O7—Na2—Na1	69.18 (4)
O1—C6—N1	119.48 (15)	O4—Na2—Na1	46.01 (3)
O1—C6—C5	123.43 (15)	O8—Na2—Na1	116.92 (4)
N1—C6—C5	117.09 (14)	O6^iii^—Na2—Na1	133.71 (4)
C8—N7—C5	102.23 (14)	O5—Na2—Na1	47.07 (3)
N7—C8—N9	116.93 (16)	Na2^iii^—Na2—Na1	152.07 (3)
N7—C8—H8	120.6 (14)	O6—Na2—Na1^iv^	61.78 (4)
N9—C8—H8	122.5 (14)	O7—Na2—Na1^iv^	145.56 (4)
C4—N9—C8	102.28 (13)	O4—Na2—Na1^iv^	130.44 (4)
C2—N10—H10A	115 (2)	O8—Na2—Na1^iv^	36.86 (3)
C2—N10—H10B	114.3 (19)	O6^iii^—Na2—Na1^iv^	88.83 (4)
H10A—N10—H10B	121 (3)	O5—Na2—Na1^iv^	86.14 (4)
O2—Na1—O8^i^	129.23 (5)	Na2^iii^—Na2—Na1^iv^	69.95 (2)
O2—Na1—O3	80.05 (5)	Na1—Na2—Na1^iv^	133.15 (2)
O8^i^—Na1—O3	80.22 (5)	O6—Na2—Na1^v^	82.59 (4)
O2—Na1—O7^ii^	81.28 (5)	O7—Na2—Na1^v^	31.10 (3)
O8^i^—Na1—O7^ii^	82.37 (5)	O4—Na2—Na1^v^	90.17 (4)
O3—Na1—O7^ii^	137.08 (5)	O8—Na2—Na1^v^	138.65 (4)
O2—Na1—O4	89.29 (5)	O6^iii^—Na2—Na1^v^	55.22 (4)
O8^i^—Na1—O4	133.29 (5)	O5—Na2—Na1^v^	139.21 (4)
O3—Na1—O4	82.51 (5)	Na2^iii^—Na2—Na1^v^	59.82 (2)
O7^ii^—Na1—O4	135.46 (5)	Na1—Na2—Na1^v^	95.369 (16)
O2—Na1—O5	133.89 (5)	Na1^iv^—Na2—Na1^v^	129.771 (19)
O8^i^—Na1—O5	90.29 (5)	Na1—O2—H2A	133 (2)
O3—Na1—O5	136.66 (5)	Na1—O2—H2B	110.3 (16)
O7^ii^—Na1—O5	82.01 (5)	H2A—O2—H2B	104 (2)
O4—Na1—O5	73.70 (5)	Na1—O3—H3A	115 (2)
O2—Na1—Na2	133.95 (4)	Na1—O3—H3B	114.2 (19)
O8^i^—Na1—Na2	92.44 (4)	H3A—O3—H3B	110 (3)
O3—Na1—Na2	90.63 (4)	Na2—O4—Na1	89.31 (5)
O7^ii^—Na1—Na2	129.14 (4)	Na2—O4—H4A	117.3 (18)
O4—Na1—Na2	44.68 (3)	Na1—O4—H4A	101.7 (18)
O5—Na1—Na2	47.31 (3)	Na2—O4—H4B	127.2 (17)
O2—Na1—Na2^i^	91.59 (4)	Na1—O4—H4B	112.0 (18)
O8^i^—Na1—Na2^i^	37.64 (3)	H4A—O4—H4B	105 (2)
O3—Na1—Na2^i^	68.95 (4)	Na1—O5—Na2	85.62 (4)
O7^ii^—Na1—Na2^i^	73.34 (4)	Na1—O5—H5C	105 (2)
O4—Na1—Na2^i^	150.82 (4)	Na2—O5—H5C	99 (2)
O5—Na1—Na2^i^	123.71 (4)	Na1—O5—H5D	96 (2)
Na2—Na1—Na2^i^	126.913 (19)	Na2—O5—H5D	148 (2)
O2—Na1—Na2^ii^	67.43 (4)	H5C—O5—H5D	111 (3)
O8^i^—Na1—Na2^ii^	74.73 (4)	Na2—O6—Na2^iii^	89.65 (5)
O3—Na1—Na2^ii^	107.25 (4)	Na2—O6—H6A	128 (2)
O7^ii^—Na1—Na2^ii^	29.94 (3)	Na2^iii^—O6—H6A	104 (2)
O4—Na1—Na2^ii^	151.97 (4)	Na2—O6—H6B	124.0 (17)
O5—Na1—Na2^ii^	110.76 (4)	Na2^iii^—O6—H6B	100.6 (17)
Na2—Na1—Na2^ii^	155.43 (3)	H6A—O6—H6B	103 (3)
Na2^i^—Na1—Na2^ii^	50.230 (19)	Na2—O7—Na1^v^	118.96 (6)
O6—Na2—O7	84.18 (5)	Na2—O7—H7A	117.7 (19)
O6—Na2—O4	166.81 (6)	Na1^v^—O7—H7A	104.2 (19)
O7—Na2—O4	83.95 (5)	Na2—O7—H7B	112.8 (18)
O6—Na2—O8	98.31 (5)	Na1^v^—O7—H7B	99.0 (18)
O7—Na2—O8	169.31 (5)	H7A—O7—H7B	101 (3)
O4—Na2—O8	94.41 (5)	Na1^iv^—O8—Na2	105.50 (5)
O6—Na2—O6^iii^	90.35 (5)	Na1^iv^—O8—H8A	119.6 (18)
O7—Na2—O6^iii^	86.16 (5)	Na2—O8—H8A	103.7 (19)
O4—Na2—O6^iii^	94.57 (5)	Na1^iv^—O8—H8B	115.2 (18)
O8—Na2—O6^iii^	83.44 (5)	Na2—O8—H8B	105.6 (19)
O6—Na2—O5	104.03 (5)	H8A—O8—H8B	106 (3)
O7—Na2—O5	108.55 (5)		

Symmetry codes: (i) −*x*, *y*+1/2, −*z*+1/2; (ii) *x*, −*y*+1/2, *z*−1/2; (iii) −*x*, −*y*, −*z*+1; (iv) −*x*, *y*−1/2, −*z*+1/2; (v) *x*, −*y*+1/2, *z*+1/2.

### Hydrogen-bond geometry (Å, º)

**Table:**

*D*—H···*A*	*D*—H	H···*A*	*D*···*A*	*D*—H···*A*
O2—H2*A*···N9^ii^	0.84 (3)	1.97 (3)	2.7875 (19)	168 (3)
O2—H2*B*···N3	0.89 (3)	2.08 (3)	2.9582 (19)	167 (2)
O3—H3*A*···O5^i^	0.87 (3)	2.08 (3)	2.9200 (18)	163 (3)
O3—H3*B*···N3	0.87 (3)	1.95 (3)	2.8038 (18)	166 (3)
O4—H4*A*···N1^vi^	0.85 (3)	1.96 (3)	2.8093 (19)	177 (3)
O4—H4*B*···N9	0.85 (3)	2.14 (3)	2.9866 (19)	176 (2)
O5—H5*C*···O1^vi^	0.81 (3)	1.96 (3)	2.7581 (18)	168 (3)
O6—H6*A*···O2^iv^	0.79 (3)	2.02 (3)	2.7938 (19)	167 (3)
O6—H6*B*···N7^vii^	0.90 (3)	2.01 (3)	2.909 (2)	173 (2)
O7—H7*A*···O1^vii^	0.88 (3)	1.95 (3)	2.7867 (17)	160 (3)
O7—H7*B*···O3	0.85 (3)	1.92 (3)	2.7608 (18)	168 (3)
O8—H8*A*···O1^vi^	0.84 (3)	1.99 (3)	2.8303 (17)	171 (3)
O8—H8*B*···N7^viii^	0.82 (3)	1.98 (3)	2.7938 (19)	171 (3)
O5—H5*D*···O1^ix^	0.78 (3)	2.02 (3)	2.7835 (17)	164 (3)

Symmetry codes: (i) −*x*, *y*+1/2, −*z*+1/2; (ii) *x*, −*y*+1/2, *z*−1/2; (iv) −*x*, *y*−1/2, −*z*+1/2; (vi) −*x*+1, *y*−1/2, −*z*+1/2; (vii) *x*−1, *y*, *z*; (viii) −*x*+1, −*y*, −*z*+1; (ix) *x*−1, −*y*+1/2, *z*−1/2.
